# Supplementary material for: Comparative Metagenomic Analysis Reveals Rhizosphere Microbiome Assembly and Functional Adaptation Changes Caused by Clubroot Disease in Chinese Cabbage
Source: Microorganisms. 2024 Jul 4;12(7):1370. doi: 10.3390/microorganisms12071370 (PMC11278620; doi:10.3390/microorganisms12071370)
Supplement: Supplementary file 1 [file microorganisms-12-01370-s001.zip › FigureS1-S9.pdf]

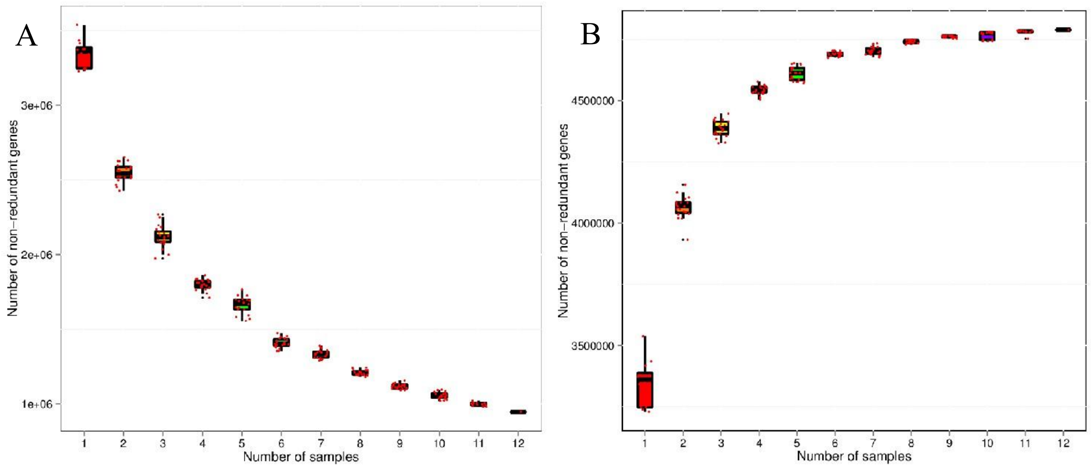

Fig. S1. The rarefaction curves of microbial core and pan genes in the rhizosphere microbiota. (A) Core gene rarefaction curve. (B) Pan-gene rarefaction curve.

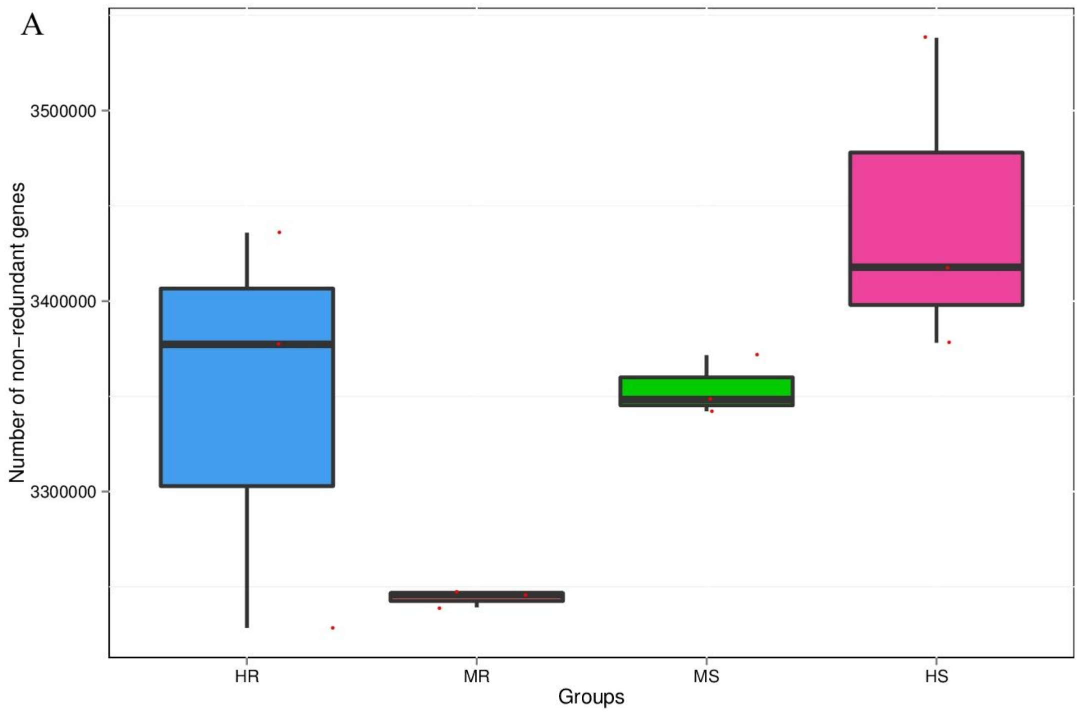

Fig. S2. Gene boxplot between different groups. HR, disease index = 0; HS, disease index ≥ 70%; MS, 30% ≤ disease index < 70%; MR, 0 < disease index < 30%.

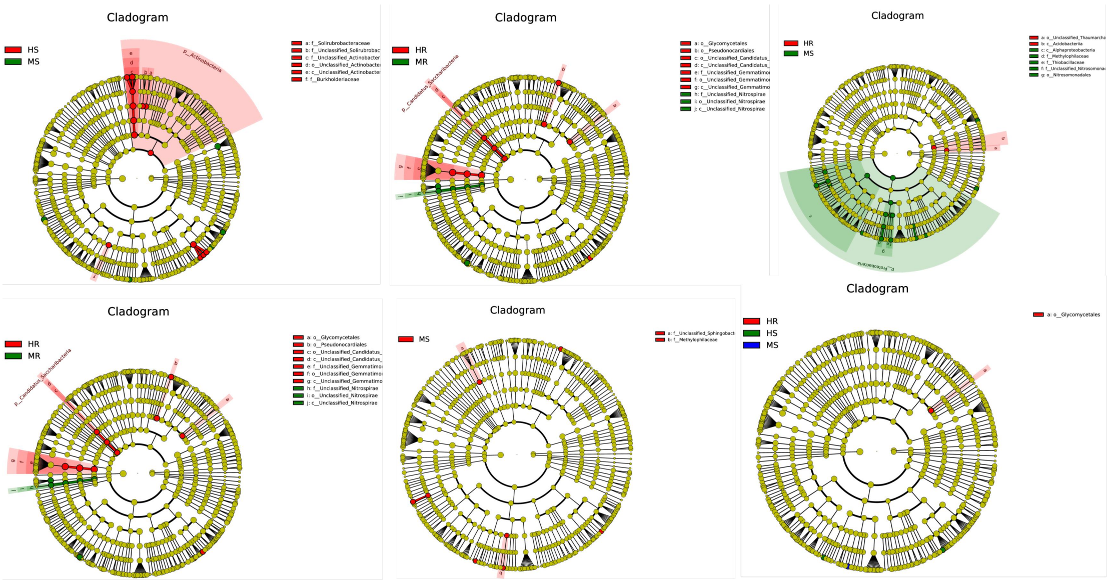

Fig. S3. The clodograms of significant differential microbial taxa between different groups in rhizosphere soils.

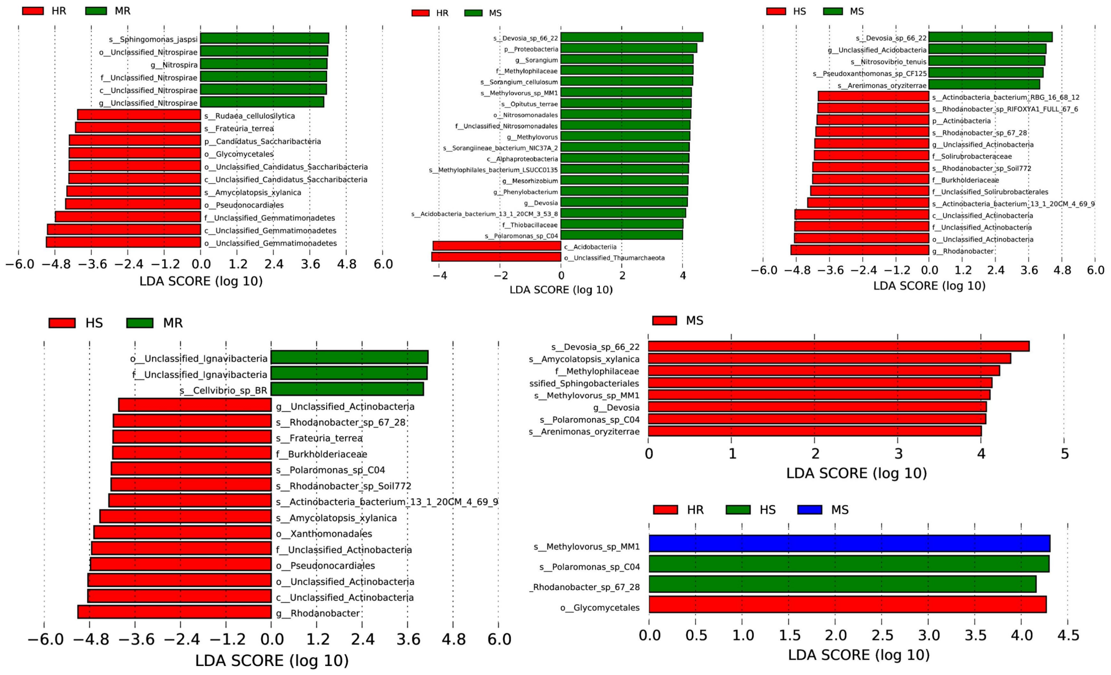

Fig. S4. Dramatic differential microbial taxa between different groups in rhizosphere soils by LEfSe analysis (LDA score>4.0, *P*<0.05).

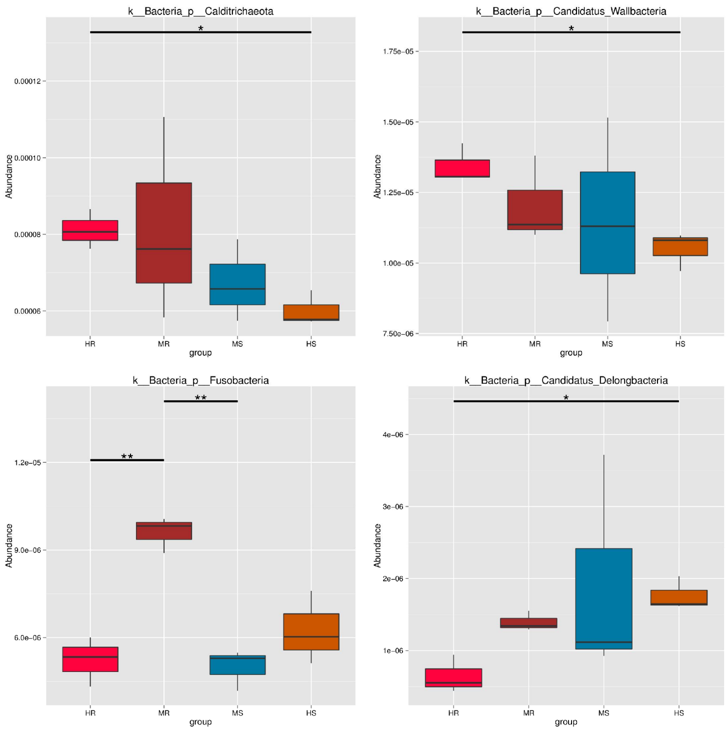

Fig. S5. Considerable different microbiomes at phylum level between different groups by MetaStats analysis.

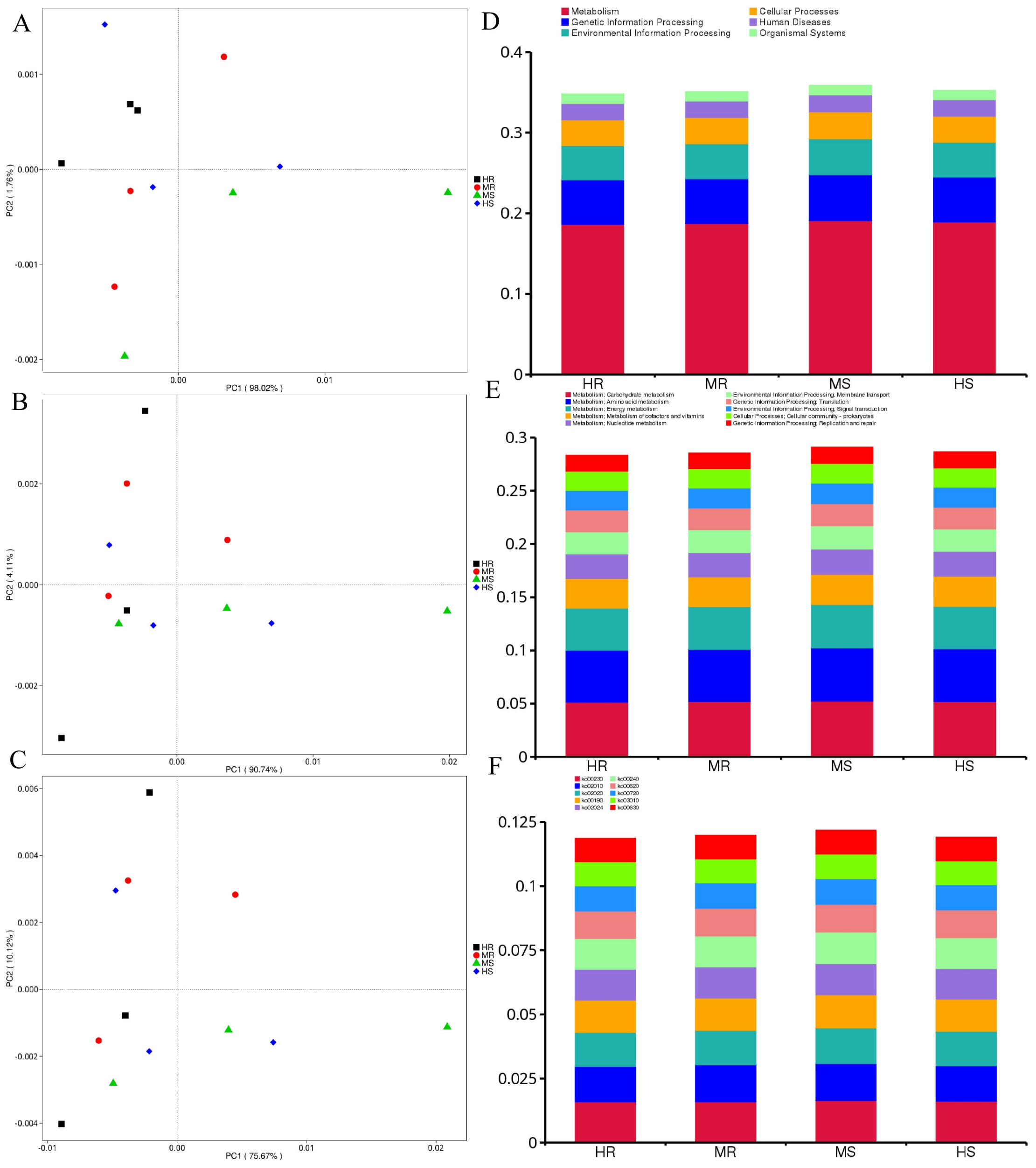

Fig. S6. KEGG functions among all samples. Principle co-ordinates analysis based on Bray-Curtis distances of microbiota descriptions in different groups at (A) level 1, (B) level 2, and (C) level 3. Relative abundances at (D) level 1, (E) level 2, and (F) level 3. HR, disease index = 0; HS, disease index ≥ 70%; MS, 30% ≤ disease index < 70%; MR, 0 < disease index < 30%.

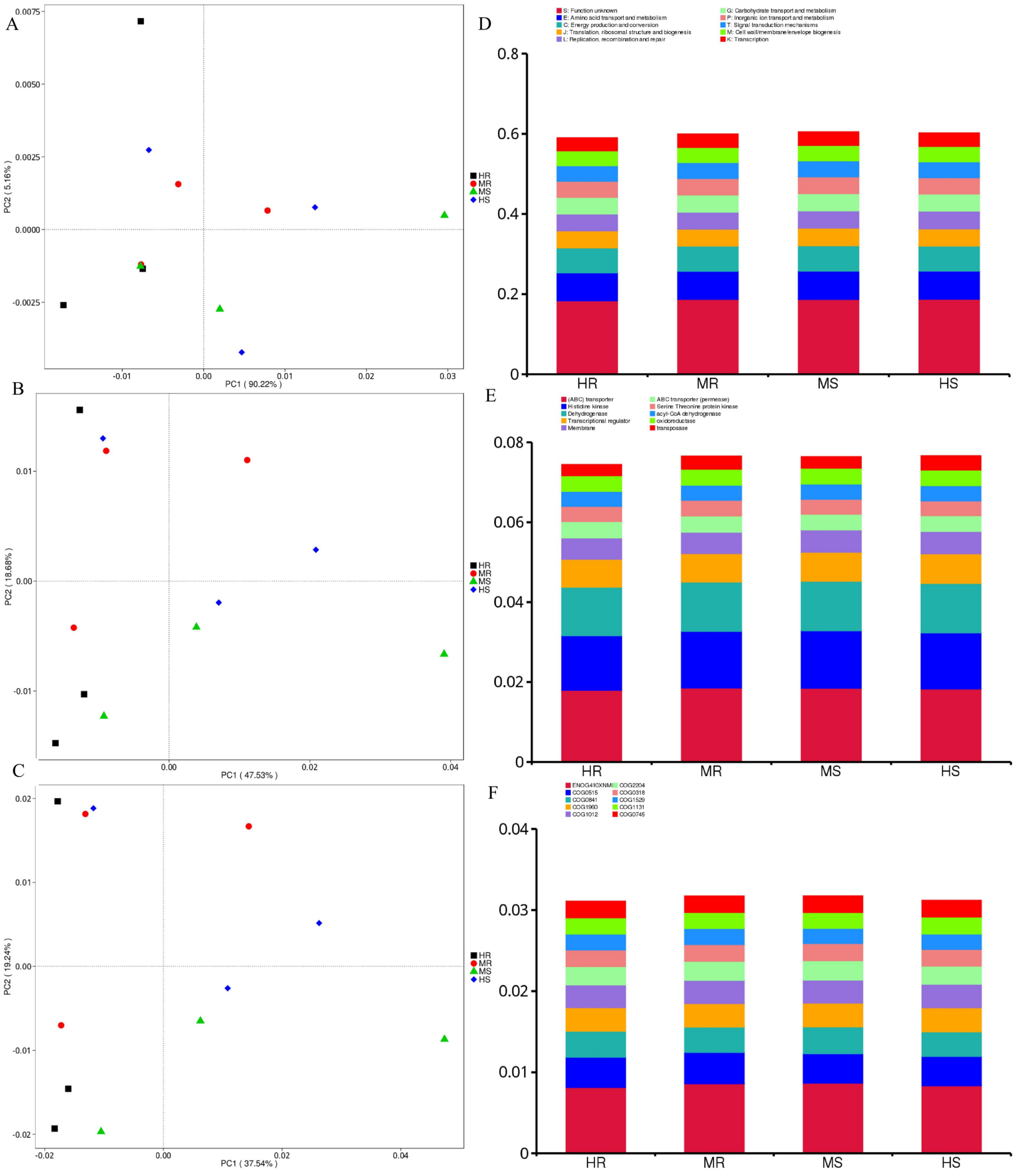

Fig. S7. Functions identified using the eggNOG database among all samples. Principle co-ordinates analysis based on Bray-Curtis distances of microbiota descriptions in different groups at (A) level 1, (B) level 2, and (C) level 3. Relative abundances at (D) level 1, (E) level 2, and (F) level 3. HR, disease index = 0; HS, disease index ≥ 70%; MS, 30% ≤ disease index < 70%; MR, 0 < disease index < 30%.

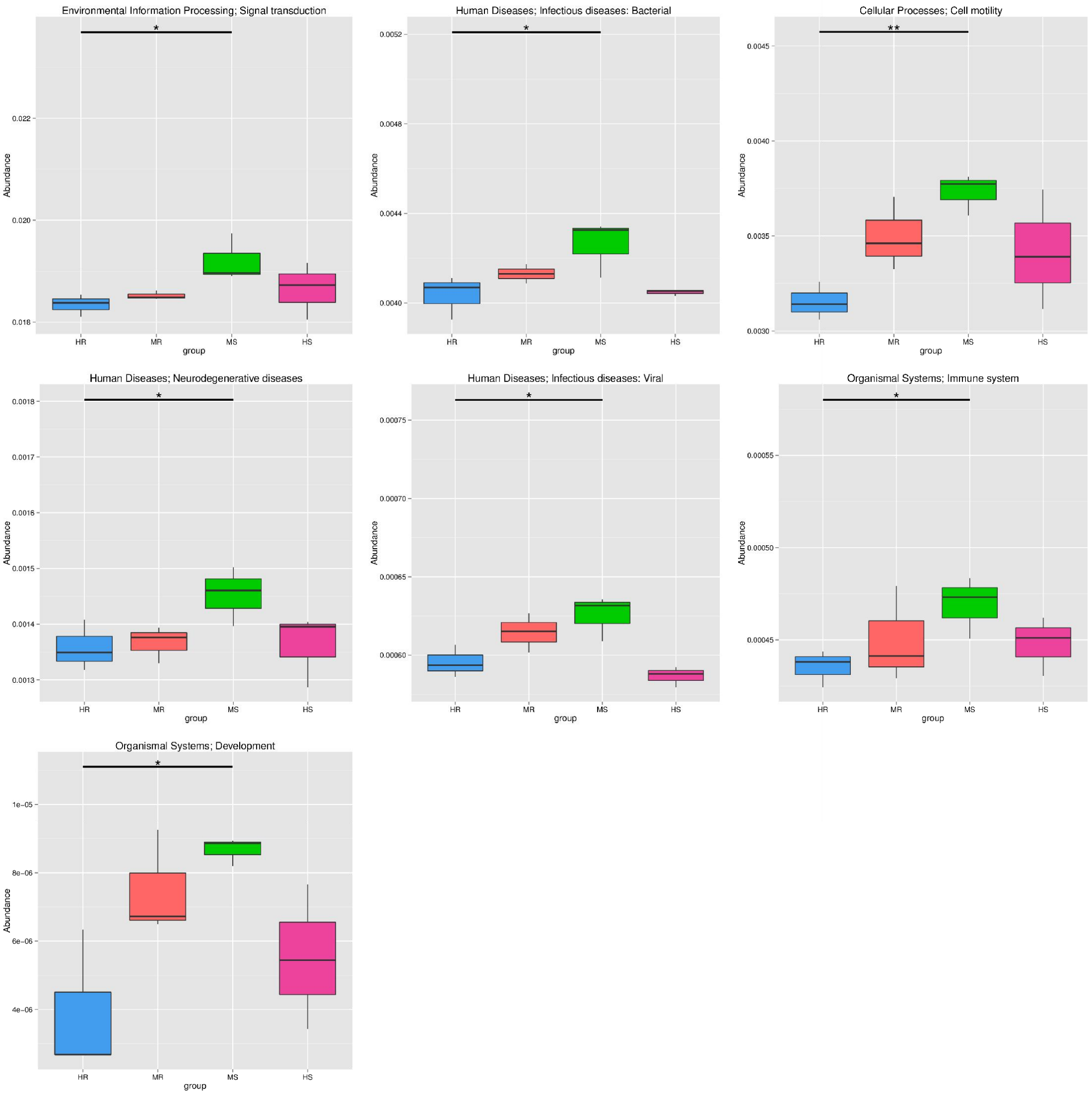

Fig.S8. The relative abundance of annotated functions with remarkable difference at level 2 using KEGG pathways.

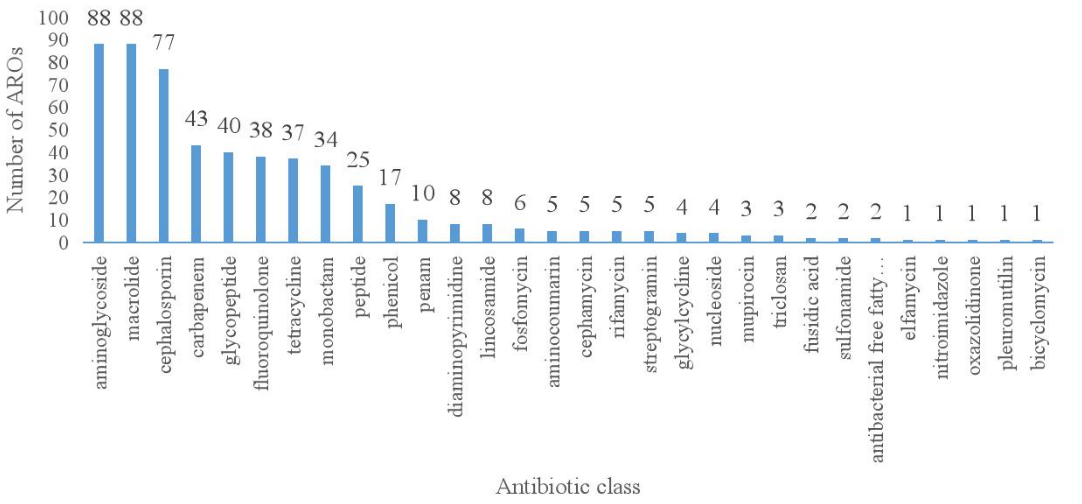

Fig.S9. Relative abundance of antibiotic classes. The numbers on the bars represent the number of AROs detected in each antibiotic class.
